# A Proteogenomic Approach to Understanding *MYC* Function in Metastatic Medulloblastoma Tumors

**DOI:** 10.3390/ijms17101744

**Published:** 2016-10-19

**Authors:** Jerome A. Staal, Yanxin Pei, Brian R. Rood

**Affiliations:** 1 Multiple Sclerosis Department, Florey Institute of Neuroscience and Mental Health, Melbourne, VIC 3052, Australia; 2 Center for Cancer and Immunology Research, Children’s National Medical Center, Washington, DC 20010, USA; ypei@childrensnational.org (Y.P.); brood@childrensnational.org (B.R.R.)

**Keywords:** medulloblastoma, MYC, quantitative proteomics

## Abstract

Brain tumors are the leading cause of cancer-related deaths in children, and medulloblastoma is the most prevalent malignant childhood/pediatric brain tumor. Providing effective treatment for these cancers, with minimal damage to the still-developing brain, remains one of the greatest challenges faced by clinicians. Understanding the diverse events driving tumor formation, maintenance, progression, and recurrence is necessary for identifying novel targeted therapeutics and improving survival of patients with this disease. Genomic copy number alteration data, together with clinical studies, identifies *c-MYC* amplification as an important risk factor associated with the most aggressive forms of medulloblastoma with marked metastatic potential. Yet despite this, very little is known regarding the impact of such genomic abnormalities upon the functional biology of the tumor cell. We discuss here how recent advances in quantitative proteomic techniques are now providing new insights into the functional biology of these aggressive tumors, as illustrated by the use of proteomics to bridge the gap between the genotype and phenotype in the case of *c-MYC*-amplified/associated medulloblastoma. These integrated proteogenomic approaches now provide a new platform for understanding cancer biology by providing a functional context to frame genomic abnormalities.

## 1. Introduction

Medulloblastoma (MB) is the most common pediatric malignant brain tumor and one of the leading causes of brain cancer deaths in children. Current therapy for high-risk medulloblastoma involves aggressive treatments that often leave survivors with significant neurological and intellectual disabilities due to the effects of these nonspecific cytotoxic therapies on the developing brain [1]. Extensive intertumoral heterogeneity is found in medulloblastoma, with at least four distinct molecular variants identified using genomic profiling techniques [2,3,4]. Significant differences in clinical outcome among these subgroups demonstrate a need for subgroup-specific therapeutic strategies, yet a better understanding of molecular drivers of disease is required before these efforts can be realized [2,5,6]. Although there is early promise for sonic hedgehog pathway (SHH) inhibitors in a subset of patients with upstream mutations [7,8], there remain few targets for the other subgroups—particularly Group 3 tumors, which have the worst overall survival rates in patients.

## 2. Group 3 Medulloblastoma and *c-MYC* Amplification

Group 3 MB remain poorly understood despite accounting for over a quarter of medulloblastoma cases and displaying significant recurrence and mortality rates (around 50% across multiple studies) [2,6]. Although transcriptional profiling analysis identifies this subgroup based on an enrichment of genes involved in GABAergic function, photoreceptor differentiation, and ribosomal biosynthesis, it fails to identify any traditional druggable signaling pathways [2,9]. Furthermore, multiple next-generation sequencing studies reveal few recurrent mutations in this subgroup except in distinct components of the epigenetic machinery (e.g., KDM6A and ZMYM3), which are shared with Group 4 tumors [4,10,11]. All studies to date, including somatic copy number analysis across 1000 medulloblastoma genomes, identify *c-MYC* copy number amplifications primarily confined to Group 3 tumors [12]. The presence of these genomic amplifications represents a high-risk group associated with poor survival, as highlighted through multivariable survival analysis of patients with Group 3 tumors [13].

## 3. Linking *c-MYC* Genomic Aberrations to Molecular Pathways Driving Tumor Behavior

**Figure 1:**
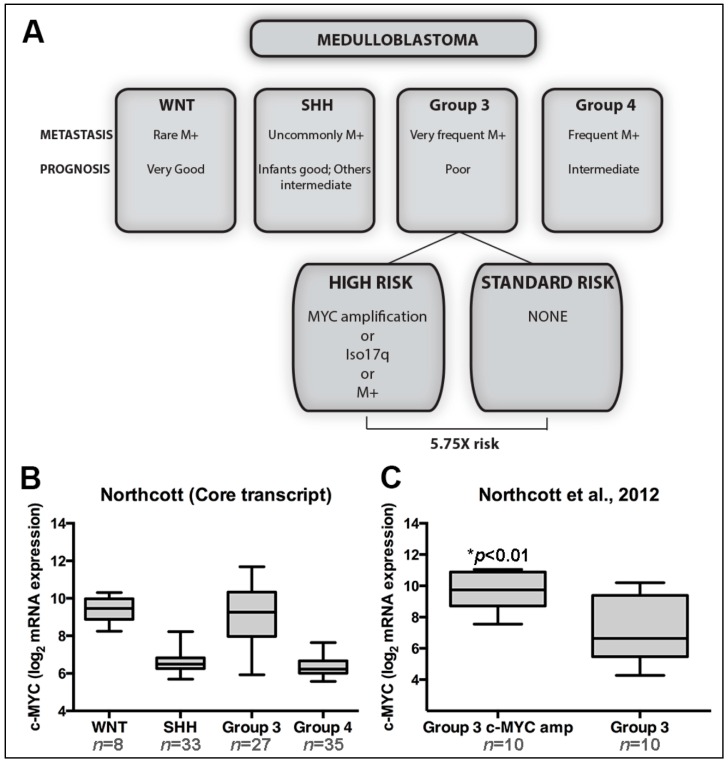
(**A**) Summary for the proposed risk stratification of MYC-amplified tumors in Group 3 medulloblastoma. Interestingly, although c-MYC genomic amplifications are almost exclusively found in Group 3 tumors and associated with poor survival (>50% survival), mRNA expression patterns are similar to that seen in the WNT subgroup (**B**) which has the best clinical outcome of all medulloblastoma subgroups. Increased gene transcripts are significantly associated with *MYC*-amplified Group 3 medulloblastomas when compared to nonamplified tumors from the same group (**C**); (Northcott et al., 2012 dataset under accession number GSE37385 [3]).

Difficulties making inferences from genomic abnormality to cancer phenotype remain problematic for all types of cancer. With regard to medulloblastoma, it is unclear how *c-MYC* amplification is driving tumor aggressiveness. Increased *c-MYC* mRNA transcripts are observed in Group 3 tumors compared to the *SHH* and Group 4 subgroups, yet there is no difference in comparison to tumors of the wingless (WNT) subgroup (Figure 1). Unlike other subgroups, WNT tumors almost never harbor any *MYC* amplifications [4,10,11] and increased transcript expression is attributed to *MYC* being a downstream target of the *WNT* signaling pathway. Further, the near-total survival of the *WNT* subgroup (Figure 1) tends to refute the idea that *c-MYC* overexpression alone is responsible for the poor survival observed in Group 3 MB. This discrepancy in prognosis has been previously highlighted in other reviews [14] and remains unresolved. Does this mean the aggressive phenotype of *c-MYC*-amplified tumors within Group 3 MB is independent of *c-MYC* expression, or that the cellular context in which *c-MYC* overexpression occurs is critical to phenotype determination? Of note, WNT medulloblastomas arise from progenitor cells in the lower rhombic lip outside the cerebellum proper [15] as opposed to Group 3 tumors which are demonstrated to originate from cerebellar stem cells or granule neuron precursors [16,17]. We do in fact see a significant difference in *c-MYC* expression levels (*p* = 0.0056; two tailed *t*-test equal variance) when we compare *MYC*-amplified and nonamplified tumors within Group 3 tumors in humans (Figure 1), suggesting that *c-MYC* copy number amplifications may indeed result in increased *c-MYC* transcript levels. In addition, amplification of homeobox proteins orthodenticle homologue 2 (*OTX2*) is also identified in Group 3 MB [12]. Moreover, *OTX2* and *c-MYC* are frequently coexpressed at high levels in medulloblastoma and regulate many of the same genes, indicating there might be a functional interaction between these two genes [18]. OTX2 is highly expressed in the developing cerebellum, playing a critical role in the regional patterning of early embryonic cells, but is silenced in adulthood. *OTX2* has recently been shown to repress differentiation, increase proliferation, and upregulate *c-MYC* in medulloblastoma cells [18,19,20].

The tumorigenic role of *c-MYC* in medulloblastoma is further complicated by RNA-Seq studies showing persistent gene fusions involving the 5′ end of *PVT1*, a noncoding gene, which frequently coamplifies with *c-MYC* [3]. In these studies, the majority of MYC-amplified tumors harbored *PVT1* fusions, which are proposed to arise as a result of chromothripsis [3]. Although *PVT1* is non-protein-coding, it is a host gene for four microRNAs, miR-1204–miR1207. Intriguingly, earlier studies have implicated miR-1204, which is expressed at a higher level in *c-MYC*-amplified Group 3 tumors compared to the adjacent miR-1205 and miR-1206, as a candidate oncogene that enhances oncogenesis in combination with *MYC* [21,22].

Taken together, the evidence supports a model in which *MYC* amplification drives an aggressive phenotype in the permissive context provided by the cell of origin and in concert with other genomic events such as *OTX2* amplification and *PVT1* fusion.

## 4. Animal Models as a Platform to Understand *c-MYC* Function in Medulloblastoma

The development of *MYC-*driven animal models of medulloblastoma has provided strong support for the role of c-MYC over-expression in the tumorigenesis of Group 3 MB. Initial independent animal models involving the induced overexpression of *c-MYC* in cerebellar stem cells or granule precursor cells have clearly shown that the resultant tumors not only display highly aggressive large cell anaplastic histology, but also are transcriptionally similar to Group 3 medulloblastoma [16,17]. These models were instrumental in identifying possible therapeutic targets such as the PI3K/mTOR [17] and histone deacetylase (HDAC) pathways. Combination targeting of PI3K/mTOR and HDAC dramatically inhibited tumor growth in preclinical mouse models and human patient-derived xenografts (PDX) (Pei et al., unpublished data under preparation for submission.). Additionally, *MYC* is not only required for tumor initiation, but also necessary for tumor maintenance. Inactivation of *c-MYC* causes rapid tumor regression in mouse model, indicating that targeting c-MYC itself might be an effective therapeutic strategy for this disease [17].

## 5. The Role of Tumor Suppressor Protein *Trp53* in Group 3 Medulloblastoma

An important caveat to the development of initial animal models for studying *c-MYC* function in Group 3 MBs is that those mentioned above have all required the loss of Trp53 function to generate *c-MYC* associated medulloblastoma phenotypes. This is particularly significant as Trp53 mutations are not common in human Group 3 MB, and their absence was proposed to be indicative of other significant cooperative genetic or molecular events that inhibit *Trp53* function, potentiating tumor initiation [14]. An intriguing new study demonstrating that *MYC* family amplifications and Trp53 pathway defects both emerged at relapse may provide support for this cooperativity [23] and may imply that loss of Trp53 function is associated with tumor progression. These additional genomic events found at relapse could not be detected at diagnosis, indicating that an acquisition of genetic or epigenetic changes might occur after initial treatment. Whether this is the case with relapsing Group 3 medulloblastoma is unclear, but new animal models of *MYC*-driven tumors without disrupted *Trp53* function are being developed and show homology with human Group 3 MB [24]. Further studies need to determine whether *MYC* plays a different tumorigenic role in these various models and whether they all result in similar tumor behavior.

In summary, although the data support *c-MYC* as a significant contributor to the phenotype and maintenance of aggressive Group 3 MB, the exact mechanisms of its contribution remain incompletely understood. This is further complicated by the fact that *c-MYC* regulates a variety of pathways, often making it difficult to determine which molecular events are driving oncogenic behavior or are simply downstream passenger events. There is a growing consensus among clinicians that the key molecular mechanisms driving the aggressive recurrence of these tumors need to be clarified in order to rationally improve therapy for these high-risk patients [25]. Thus, MYC biology provides a case study for the most important challenge facing translational research in the genomic era. Given the incredible breadth of genomic output, there is a tremendous challenge in determining which events are driving malignant biology and thus should be the focus of therapeutic intervention. One concept that can provide insight into this issue is the tenet that genomic events driving biology should project into the cellular proteome, which comprises the bulk of the functional molecules of the cell.

Significant developments are being made in proteomic techniques and these are now providing clinicians with unique insights into commonly studied cancers [26]. Researchers are realizing the importance of proteomics as a platform to link the genome to phenotype, and a substantial investment has been made in developing our understanding of the human proteome (see the draft map of the human proteome project [27]). These techniques are also now providing promising new insights into the study of *c-MYC*-amplified Group 3 medulloblastomas.

## 6. Advances in Proteomic Profiling of Cancers

Protein profiling in cancer has advanced tremendously from early techniques, almost half a century ago, when starch gel electrophoresis was being used to determine globulin expression profiles in serum from patients with myeloma and macroglobulinemia [28]. Possibly one of the greatest advances in protein profiling was the introduction of polyacrylamide gels and isoelectric focusing in the early 1960s that helped introduce two-dimensional polyacrylamide gel electrophoresis (2D-PAGE) and allowed multiple numbers of proteins to be studied simultaneously. 2D-PAGE studies have been extensively utilized in a wide range of studies covering nearly every cancer type [29]. The ongoing research using 2D-PAGE has yielded significant results over the past decades and helped identify several proteins as candidate prognostic markers of particular cancers [29,30]. Despite improvements in the 2D-PAGE approach, including fluorescence difference gel electrophoresis (DIGE), this technique is often associated with a limited dynamic range, making it difficult to profile low-abundance proteins [31]. Proteomic profiling using mass spectrometry, on the other hand, continues to gather momentum, in large part due significant advances in instrumentation. The development of matrix-assisted laser desorption ionization (MALDI) MS, for example, played a pivotal role in protein expression maps of lymphoid neoplasms [32], and together with electrospray ionization (ESI), MS has allowed the quantitative analysis of increasing numbers of proteins from a variety of cancers [33]. The next generation of liquid chromatography mass spectrometers (LC–MS), with increased sensitivity and speed, together with high-resolution isoelectric focusing (HiRIEF), enables deep proteome coverage and unbiased proteogenomic studies of cancer tissues [34].

Major concerns regarding proteomic techniques stem from the limited depth and reproducibility of some of the early MS technologies. However, there have been significant contemporary multi-laboratory collaborative studies, sponsored by the U.S. National Cancer Institute (NCI) and the Human Proteome Organization (HUPO), which have examined the sources of irreproducibility in MS-based proteomics [35,36]. These studies have demonstrated the high reproducibility across laboratories and instrument platforms of MS-based assays of proteins in low microgram per milliliter concentrations [35]. At present, optimized in-depth profiling techniques can identify proteins in biological samples that span six or more logs of protein abundance [37]. Furthermore, great steps have been made in not only identifying large numbers of proteins, but also in the ability to accurately and reliably quantitate them. Currently considered the gold-standard for proteomic biology discovery, metabolic stable isotopic labeling by amino acids in cell culture (SILAC) provides an accurate means for quantitative protein analysis and has been extensively used in cell- and tissue-based proteomics analyses.

## 7. Protein Quantification Using Stable Isotopic Labeling by Amino Acids in Cell Culture

SILAC was first described almost 10 years ago and is one of the most popular peptide labeling techniques to date. The general concept is that relative and absolute quantification of a sample of interest can be performed when comparing its MS intensity with that of a labeled peptide standard introduced into the sample. To create the standard, peptides are metabolically labeled in culture by introducing the isotope label into every protein during cell growth and division, generating a labeled standard for every protein. This is done by growing parallel populations of the same cell line, one in media containing a “light” (normal) amino acid and the other in media containing a “heavy” amino acid. The heavy amino acid can contain a ^13^C instead of ^12^C, an ^15^N instead of ^14^N, or ^2^H instead of H. When incorporated into a peptide, the heavy amino acid leads to a known mass shift (for example, 6 Da in the case of ^13^C_6_-Arg) compared to the peptide with the normal (light) version of the amino acid [38]. In a simple experimental scheme, protein lysates from the two cell lines (heavy and light) are mixed in equal amounts and their proteomes are measured using MS. A pair in the mass spectra will occur for each peptide with the lower mass originating from the unlabeled sample and quantified as a relative ratio to that of the heavy corresponding mass spectra, which originated from the labeled cell line. When the heavy and light cell lines are exposed to different experimental conditions (e.g., hypoxia and normoxia), the resulting alterations in the proteome can be measured. In a more translational setting, SILAC has been used to compare microsomal fractions of more metastatic versus less metastatic prostate-cancer cell lines [39], to identify novel prognostic markers of breast cancer progression [38], and to quantify N-linked glycosylation in diffuse large B-cell lymphoma subtypes [40,41].

**Figure 2:**
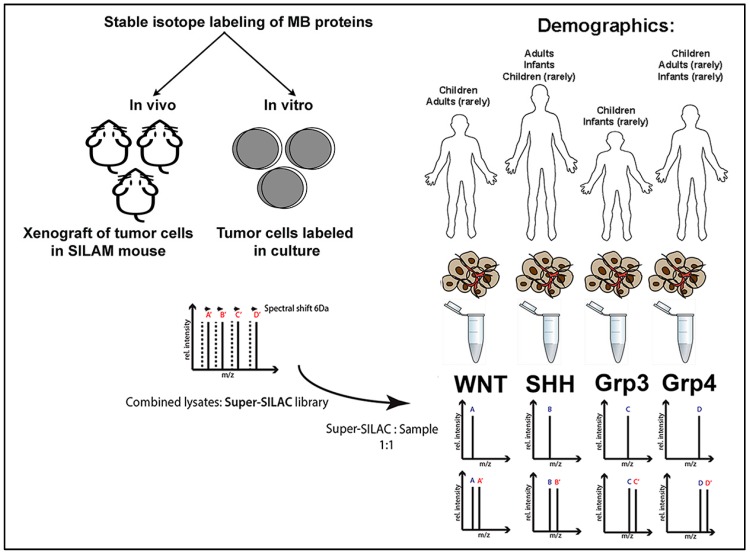
Schematic presentation of super-SILAC (stable isotopic labeling by amino acids in cell culture) methodology. All proteins within different medulloblastoma primary/cell lines are isotopically labeled, in vivo (using stable isotope labeling of amino acids in mammals: SILAM) or in vitro, through incorporation of ^13^C_6_-Arg (MB—medulloblastoma). This causes a known shift of 6 Da in the mass spectrometry spectra. The combined lysates of the isotopically labeled cultures are mixed (1:1 ratio) with tumor lysates from different medulloblastoma tumor/cell samples resulting in two spectra profiles per protein (separated by 6 Da). The protein of interest can now be quantified in the sample as a ratio of the isotopically labeled protein in the same sample.

An extension of the SILAC protocol has been the creation of a reference standard from a mixture of multiple SILAC-labeled cell lines (termed super SILAC) that better serves for quantifying proteins (Figure 2) in complex tissues [38]. The use of a multiply sourced SILAC-labeled protein standard increases quantification accuracy, as the combined SILAC reference better represents the total proteome of a given tumor tissue despite expected intratumoral heterogeneity [38]. However, although this method offers an ideal platform for large-scale relative quantification studies in tissue samples, the limitations lie in the need to produce a reference standard that ensures significant and adequate proteome coverage of all the proteins found in the tissue to be analyzed. Partially offsetting this disadvantage is that the quantitated proteins must be present in both the reference standard and the tumor tissue and are thereby necessarily tumor cellular proteins and not proteins derived from contaminating stromal elements; this feature improves the signal-to-noise ratio.

## 8. Proteomic Analysis of Medulloblastoma and c-MYC Function

Early proteomic profiling studies in medulloblastoma focused on biomarker identification in cerebrospinal fluid (CSF) samples from pediatric patients [14,42]. Possible host responses to tumor presence were detected when comparing tumor versus control samples using 2D-PAGE followed by MALDI-time of flight MS analysis [42]. Independent studies using the same proteomic analysis technique further highlighted the power MS-based proteomics to stratify surgical medulloblastoma tissue samples based on histotype (classic versus desmoplastic/nodular) [43] or to study cancer stem cell development within cultured medullospheres [44]. Not surprisingly, current research is rapidly moving towards integrated genomic, bioinformatic, and proteomic techniques, which has recently been used to provide novel insights into functional and signaling pathways in medulloblastoma tumors harboring a 17p deletion and diffuse intrinsic pontine gliomas [45,46]. Recent work within our group highlights the potential of new quantitative MS-based techniques to uncover the molecular pathways and possible genetic drivers of *c-MYC* function in Group 3 medulloblastoma [47]. Taking a super-SILAC-based approach, we constructed a novel medulloblastoma-specific reference standard that demonstrated impressive quantification accuracy and efficiency when measuring proteins from tumor cells across all the medulloblastoma subgroups. Distinct protein expression patterns between *c-MYC* amplified versus nonamplified tumors were observed, suggesting significant metabolic pathway differences between these groups [47].

Our proteomic results showing increased expression of ribonucleoproteins (HNRNPs) associated with spliceosome activity (HNRNPC, HNRNPA1, HNRNPA2, and PTB) is supported by earlier work looking for c-MYC-dependent proteins in the medulloblastoma cell line D425Med [48]. Furthermore, these proteome differences also partially overlap with those seen in the medulloblastoma transcriptome studies [3]. Although never studied in the context of medulloblastoma, *c-MYC* is known to regulate the expression of these proteins [49]. Importantly, these HNRNPs influence downstream metabolism-related pathways through the altered expression of the pyruvate kinase M2 (PKM2) isoform, which was also observed in our study, resulting in the Warburg effect. This may provide a competitive advantage to the tumor cells by allowing increased survival under hypoxic and nutrient poor conditions. Together, these proteomic results provide functional insight into *c-MYC*-driven tumors and may help to explain the metastatic propensity of these tumor subtypes.

## 9. Characterizing Medulloblastoma Using MicroRNA–Proteomic Networks

MicroRNAs (miRNAs) are essential regulatory factors in the development of the central nervous system and differentially expressed across different medulloblastoma subtypes [50,51]. Early studies identified specific miRNA expression patterns that could distinguish differing medulloblastoma molecular features, such as *c-MYC* overexpressing tumors, and may therefore provide interesting insights into the factors regulating tumorigenesis [51]. Yet there is little known regarding how miRNAs influence medulloblastoma tumor behavior.

A recent study conducted a specific analysis of proteins, miRNAs, and genes to characterize human medulloblastoma stem-like cells (hMB-SLCs) [52]. These hMB-SLCs represent a significant proportion of the tumor cell population and are associated with a poor prognosis [53]. Pathway analysis (using the Genomatix Pathway System) of differentially expressed miRNAs together with deregulated genes and proteins revealed exciting new subnetworks involved in the maintenance of hMB-SLCs, including core pluripotency factors such as *OCT4* and *KLF4* [52]. Similarly, another study showed that miRNA biogenesis regulates an important developmental transition in granule cells of the cerebellum via the Sonic Hedgehog (Shh)–Patched (Ptch) signaling pathway, which significantly alters medulloblastoma growth phenotypes [54].

Emerging studies clearly highlight the differential expression of miRNAs in different medulloblastoma subgroups, and proteomic analysis is providing key insights into how these factors may be regulating specific oncogenic pathways. These are particularly important when dealing with genomic aberrations that occur in non-gene-coding regions, such as seen with *PVT1–MYC* gene fusions in Group 3 medulloblastomas [3]. Integrating miRNA–proteomic networks into our current understanding of genomic abnormalities therefore provides a clearer picture of functional pathways regulating tumor behavior and may refine future therapeutic interventions.

## 10. Proteomic Analysis of Medulloblastoma-Derived Exosomes

Exosomes are membrane-derived extracellular vesicles that are shown to influence various cellular interactions. Intriguingly, tumor cells produce exosomes that can significantly influence a number of cancer-related processes such as metastatic invasion, drug resistance, angiogenesis and cellular growth [55]. Exosomes serve as an alternative intercellular trafficking mechanism that enables exchange of molecules, including oncogenic proteins and nucleic acids, that could be susceptible to extracellular degradation if released via classical secretory pathways. Initial studies demonstrated the exosome-mediated transfer of biologically active oncogenic EGFRvIII proteins from glioma cells into culture media and plasma of tumor xenograft-bearing mice [56]. Similarly, elevated c-MYC RNA was found in exosomes isolated from mice harboring human medulloblastoma xenotransplants [57]. Cultured medulloblastoma cells with amplified *c-MYC* had higher DNA/RNA levels of this oncogene within exosomes compared to non-*c-MYC*-amplified cells [57]. It is suggested that exosomes isolated from plasma or CSF of medulloblastoma patients may be used to decipher molecular features underlying malignancy or reflect responses to therapy [57].

Proteomics is a powerful tool to identify the functional role for exosomes in medulloblastoma. Initial studies characterized the structure and expression of exosomal proteins from the medulloblastoma cell line D283MED, and further demonstrated increased tumor cell proliferation and migration when these exosomes were added to medulloblastoma cell lines [58]. The transcription factor hepatocyte nuclear factor 4 α (HNF4A) was identified as having a possible role as a tumor suppressor in this this cell line using pathway analysis of the exosomal proteins [58]. Subsequent examination of these initial proteomic results, focusing on transcriptional regulators, found further proteins that significantly influence downstream, cancer-related pathways (e.g., BRCA1, p53) [59].

It is abundantly clear that multiple post-transcriptional factors significantly restrict direct correlations between genomic aberrations and resultant oncogenic behavior. In addition to this, exciting new research now reveals the significance of extracellular vesicular release (exosomes) in modulating cancer cell behavior, either through altering transcriptional pathways or directly through proteomic signaling mechanisms. In this regard, proteomics is shedding new light into the influence of miRNAs and the other exosomal components on tumor cell behavior and response to injury.

## 11. Conclusions

Quantitative proteomics has undergone rapid technological improvement over the past decade. The majority of this growth has occurred in the context of tightly controlled experimental variables and has not yet been extensively tested in the arena of clinical tissue samples or biofluids. Applied proteomics carries the promise of visualizing the functional compartment of the cell and thus the interface of cellular biology and cellular bioprocesses helping to focus on the link between genetic changes and functional phenotypes and to identify potential therapeutic targets. Additionally, this compartment is rich in therapeutic opportunities. For example, studies of protein modifications, such as phosphoproteomics offer the ability to understand active signaling matrices and compartment-based proteomics (e.g., membrane proteins) can identify antigens for targeted antibody- and cell-based immunotherapies. This approach provides a platform for understanding the biology of other cancers by providing a functional context to frame genomic abnormalities.

Currently, the proteomics field struggles with some significant challenges such as the lack of bona fide housekeeping proteins for intercellular normalization, the dynamic range of detection necessary to quantitate the entire proteome across its naturally occurring abundance range, and the lack of sensitivity necessary to tackle intercellular tumor heterogeneity or rare biomarker detection (e.g., peptides resulting from signature coding mutations). In cancer research, genomic discovery platforms are remarkably capable, yet the data they provide is removed from the functional biology they ultimately control. This fact creates a challenge in contextualizing the massive yield of findings. Viewing the genomic landscape through the lens of proteomics may provide the necessary context by identifying which genomic aberrations are translated to the functional compartment of the cell, thus helping to discern the essential genomic features of a tumor. A rapid expansion of applied quantitative proteomic capabilities is anticipated in the near future with implications for biological investigation, drug target identification, and biomarker discovery.
